# A novel NKX2-1 frameshift variant expanding the genetic landscape of benign hereditary chorea

**DOI:** 10.1007/s10072-025-08697-4

**Published:** 2025-12-22

**Authors:** Simone Aloisio, Martina De Riggi, Adriana Martini, Sofia Grandolfo, Daniele Birreci, Luca Angelini, Bianca Laura Cinicola, Alberto Spalice, Matteo Bologna

**Affiliations:** 1 Department of Human Neurosciences, Sapienza University of Rome, Viale dell’Università, 30, Rome, 00185 Italy; 2 Department of Translational and Precision Medicine, Sapienza University of Rome, Rome, Italy; 3 IRCCS Neuromed, Pozzilli, Italy; 4 Department of Maternal and Child Health and Urogynecological Sciences, Sapienza University of Rome, Rome, Italy

**Keywords:** NKX2-1, TTF-1, Benign hereditary chorea, Pediatric movement disorders, Frameshift variant

## Abstract

**Introduction:**

Benign hereditary chorea (BHC) is a rare childhood-onset hyperkinetic disorder caused by pathogenic variants in NKX2-1. It typically follows a stationary or only mildly progressive course and may be associated with thyroid and respiratory involvement, constituting the “brain–lung–thyroid” syndrome.

**Methods:**

We report the case of a girl carrying a novel heterozygous NKX2-1 frameshift variant. Clinical assessment included longitudinal neurological examinations, neuroimaging, laboratory testing, cardiological evaluation, and genetic analysis.

**Results:**

A 6-year-old girl, first evaluated at age 3 for delayed psychomotor milestones, showed choreiform movements with motor impersistence, milkmaid’s grip, and imbalance, sparing the forehead. Brain MRI was normal. Thyroid dysfunction was documented since foster care, while no respiratory involvement was reported. Genetic testing in August 2024 identified a novel heterozygous NKX2-1 frameshift variant (c.246_250del p.Met83AlafsTer354), classified as likely pathogenic. At last follow-up, movements persisted but remained non-progressive.

**Discussion:**

This case expands the mutational spectrum of NKX2-1-related disorders. The stationary course of chorea, together with thyroid involvement and absence of pulmonary disease, aligns with previous literature. Recognition of novel NKX2-1 variants is essential to improve genotype–phenotype correlations, avoid misdiagnosis with progressive pediatric choreic or ataxic syndromes, and provide accurate prognostic counselling, as most affected individuals achieve a favorable long-term functional outcome.

The online version contains supplementary material available at 10.1007/s10072-025-08697-4.

## Introduction

Benign hereditary chorea (BHC) is a rare childhood-onset hyperkinetic disorder, classically non-progressive and often improving in adolescence or adulthood [1]. It belongs to the spectrum of NKX2-1-related disorders, also known as the “brain–lung–thyroid syndrome” [2]. The NKX2-1 gene (also referred to as thyroid transcription factor 1, TTF-1) encodes a homeobox-containing transcription factor that plays a pivotal role in embryonic development and functional maintenance of multiple organs [3]. In the thyroid, NKX2-1 regulates genes critical for hormone biosynthesis; in the lung, it modulates surfactant protein expression; and in the central nervous system, it orchestrates the differentiation of specific neuronal populations within the basal ganglia and hypothalamus. Pathogenic variants in NKX2-1 typically act through haploinsufficiency, resulting in a broad phenotypic spectrum ranging from isolated non-progressive chorea to congenital hypothyroidism and respiratory disease [3]. Over the past two decades, several case reports and series have described different types of NKX2-1 mutations—including missense, nonsense, frameshift, splice-site changes, and large deletions—associated with BHC and related phenotypes (Table 1). Despite these observations, robust genotype–phenotype correlations remain elusive: the same variant may lead to variable clinical manifestations even within the same family, and early truncating variants, although sometimes linked to more complex presentations, do not invariably predict disease severity. Given this background, we report a novel NKX2-1 frameshift variant identified in a pediatric patient with BHC. This case expands the mutational spectrum associated with the disorder and contributes to the growing evidence on the genotypic and phenotypic variability of NKX2-1–related disease.

**Table 1:** Reported NKX2-1 variants and associated clinical phenotypes

Ref.	Study	Type of study	Major findings: NKX2-1 variant/phenotype
1	Breedveld et al., 2002	Case series	79-bp deletion exon 3 (p.Ser330fsX24); frameshift distal. Benign hereditary chorea; variable severity intrafamilial; normal MRI
7	Mahajnah et al.,2007	Case report	c.348del (p.Cys117Alafs*8); frameshift early. Chorea + dystonia; hypothyroidism; DBS used in one case
3	Inzelberg et al., 2011	Case report	c.623G > T (p.Trp208Leu); missense, homeobox domain. Benign hereditary chorea, non-progressive; good long-term outcome
4	de Gusmao et al., 2016	Case report	c.524 C > A (p.Ser175*); nonsense early truncation. Chorea, hypotonia, ataxia, dystonia; congenital hypothyroidism
Present case	Aloisio et al.	Case report	c.246_250del (p.Met83AlafsTer354); frameshift early. Childhood-onset chorea, motor impersistence, hypothyroidism; stable course

## Methods

We report the case of a 6-year-old female carrying a heterozygous NKX2-1 frameshift variant, presenting with developmental delay and choreiform movements. Clinical evaluation included a detailed neurological examination and follow-up visits. Neuroimaging with brain MRI, laboratory testing, cardiological assessment, and genetic analysis were performed to characterize the phenotype and exclude alternative etiologies.

## Results

The patient, currently a 6-year-old caucasian female with no available family history due to foster care placement, was first evaluated at the age of 3 years for delayed psychomotor milestones. She was able to sit unsupported at around 10 months and began to walk independently at 19 months, while she spoke her first words at 20 months. At the time of admission, she was undergoing weekly speech therapy and psychomotor rehabilitation, which are still ongoing. Thyroid dysfunction, treated with levothyroxine, was documented since her arrival in foster care, although the exact onset is undetermined. No respiratory problems have been reported, although lack of detailed information from the first years of life represents an anamnestic limitation. The first neurological evaluation, performed in October 2022 when the girl was 3 years old, revealed sudden, erratic, and random movements, including facial grimacing, both truncal and appendicular, unpredictable and purposeless in nature. These movements, which according to her foster caregivers had appeared shortly before, spared the forehead. They increased during cognitive and motor tasks and were not suppressible. A positive milkmaid’s grip and motor impersistence were present, with imbalance attributable to the involuntary movements, while the remainder of the neurological examination was unremarkable, including smooth pursuit and saccadic eye movements. Brain MRI (July 2023) was normal. Cardiological evaluation showed only sinus arrhythmia appropriate for age. Routine laboratory testing revealed elevated TSH consistent with subclinical hypothyroidism. Genetic analysis, performed in August 2024, identified a heterozygous NKX2-1 variant, c.246_250del p.(Met83AlafsTer354) (Table 2). This frameshift alters the protein sequence from residue 83, abolishes the physiological stop codon at position 401, and results in an elongated protein with an additional 34 amino acids at the C-terminus. According to ACMG criteria, the variant was classified as likely pathogenic. At the latest follow-up (August 2025), the girl remained neurologically stable, with persistent but non-progressive involuntary movements and slight imbalance (Video 1).

**Table 2:** Genetic analysis

**Genetic Finding:**
NKX2-1 14q13.3, c.246_250del p.(Met83AlafsTer254), Heterozygosis, potentially pathogenic variant

## Discussion

This case illustrates the rich phenomenology of NKX2-1-related disorders. Our patient displayed several core features consistently reported in the literature: early hypothyroidism, female predominance, delayed motor milestones, and chorea [4]. The absence of pulmonary involvement is not unexpected, as brain–thyroid–lung manifestations occur in approximately 36% of cases, brain–lung in 10%, and isolated brain involvement in 21%. BHC is typically stationary or only minimally progressive [4]. Since its first descriptions, it has been distinguished from neurodegenerative choreic syndromes such as Huntington’s disease by its childhood onset, non-progressive course, and lack of cognitive decline [5]. Long-term follow-up studies indicate that chorea often stabilizes or even improves in adolescence or early adulthood, with many patients achieving functional independence despite persistent mild motor abnormalities [6]. More recent systematic reviews confirm that delayed motor milestones and choreiform movements are the most frequent neurological features, while intellectual disability remains uncommon [3]. From a genetic perspective, the spectrum of NKX2-1 pathogenic variants is broad, including missense, nonsense, frameshift, splice-site mutations, and larger deletions. Frameshift and nonsense variants typically act through haploinsufficiency, while missense variants, particularly within the homeobox domain, may retain partial function. Reported phenotypes range from isolated non-progressive chorea to the full “brain–lung–thyroid” syndrome. For instance, frameshift variants such as p.Cys117Alafs8 and nonsense mutations like p.Ser175 have been associated with chorea combined with dystonia, ataxia, and congenital hypothyroidism, while missense variants within the homeobox domain (e.g., p.Trp208Leu) have been linked to non-progressive chorea with good long-term outcomes. Distal frameshift mutations (e.g., p.Ser330fsX24) have been reported in familial cases of BHC with intrafamilial variability. A detailed overview of reported variants and their phenotypic correlates is provided in Table 1. Despite these observations, robust genotype–phenotype correlations remain elusive. The same variant may cause strikingly different clinical pictures within or across families, underscoring the role of additional genetic, epigenetic, or environmental modifiers. In general, early truncating variants tend to be associated with more complex phenotypes, while missense mutations in the homeobox region often lead to milder, chorea-predominant presentations; however, exceptions are frequent, and clinical variability remains the rule rather than the exception. In this context, the identification of a novel NKX2-1 frameshift mutation (c.246_250del p.Met83AlafsTer354) further supports the role of loss-of-function variants as pathogenic drivers of BHC. The mutation is predicted to disrupt the protein structure, extend the C-terminal tail, and abolish the physiological stop codon, consistent with haploinsufficiency. Given the absence of previous reports in the literature or databases, this case broadens the mutational landscape of NKX2-1 and reinforces its link to isolated choreic phenotypes with thyroid involvement but no pulmonary disease. From a clinical perspective, awareness of the stationary course of BHC is crucial to avoid misdiagnosis with progressive pediatric movement disorders such as ataxia telangiectasia, neurodegeneration with brain iron accumulation (NBIA) - particularly PKAN - or juvenile Huntington’s disease [3]. In our case, the lack of progression observed during the 3-year follow-up period should be interpreted with caution, as a longer observation may be required to confirm the stable nature of the disease course. Accurate genetic diagnosis allows appropriate counselling, reassuring families about the generally favorable prognosis and guiding therapeutic choices, which are mainly supportive and rehabilitative. At present, no specific therapy is available for BHC, although sporadic reports describe symptomatic improvement with methylphenidate or corticosteroids, but evidence remains anecdotal [7]. In conclusion, our case adds to the growing body of evidence supporting the association between NKX2-1 mutations and benign hereditary chorea. Reporting novel variants remains essential to refine genotype–phenotype correlations and to better predict disease trajectory, which in the majority of patients is stationary and compatible with a favorable long-term functional outcome.

## Supplementary information

Below is the link to the electronic supplementary material.

Supplementary File 1 (mp4. 46.7 MB) **Video 1. **Main clinical features of a 6-year-old caucasian female carrying the heterozygous NKX2-1 c.246_250del p.(Met83AlafsTer354) variant, presenting with childhood-onset chorea characterized by sudden, erratic, and purposeless involuntary movements of the trunk, limbs, and face (sparing the forehead), associated with motor impersistence, in the absence of ataxia or dystonia.
